# Markedly Elevated CA 19‐9 in ANA/ASMA‐Negative IgG4‐Related Autoimmune Hepatitis With Concomitant Pancreatitis: A Case Report

**DOI:** 10.1155/crgm/3494971

**Published:** 2026-04-19

**Authors:** Hunter Scott, Samuel Reiss

**Affiliations:** ^1^ Department of Internal Medicine, Cleveland Clinic, Cleveland, Ohio, USA, clevelandclinic.org

**Keywords:** autoimmune hepatitis, autoimmune pancreatitis, CA 19-9, case report, IgG4-related disease

## Abstract

**Background:**

IgG4‐related disease (IgG4‐RD) can involve the liver and pancreas and may present without conventional autoimmune hepatitis (AIH) autoantibodies, creating diagnostic delay unless IgG4 testing and tissue immunostaining are pursued. We describe an older man with acute hepatocellular injury and marked hyperbilirubinemia who was negative for antinuclear (ANA) and antismooth muscle (ASMA) antibodies but had markedly elevated serum IgG4 and IgG4‐positive plasma cell infiltration on liver biopsy, consistent with IgG4‐related autoimmune hepatitis (IgG4‐AIH).

**Case Presentation:**

A man in his 80s presented with abdominal pain followed by progressive jaundice and pruritus, with acute hepatocellular injury and hyperbilirubinemia. Evaluation showed negative ANA and ASMA, elevated total IgG (2166 mg/dL) and IgG4 (442.5 mg/dL), CA 19‐9 1268 U/mL, and CT/MRCP findings consistent with pancreatitis without biliary obstruction or mass. HFE analysis showed compound heterozygosity. Liver biopsy demonstrated interface hepatitis with plasma cell–rich inflammation and increased IgG4‐positive plasma cells (up to 58 per high‐power field), supporting IgG4‐AIH. Minimal iron deposition on biopsy favored inflammation‐related iron study abnormalities rather than primary hemochromatosis. The pancreatic findings were most consistent with probable Type 1 autoimmune pancreatitis. Prednisone 40 mg daily led to improvement, with normalization of transaminases and total bilirubin by Day 57 (approximately 8 weeks) in available follow‐up testing.

**Conclusion:**

IgG4‐AIH is diagnostically challenging, particularly in seronegative presentations. Unexpectedly high CA 19‐9 may be seen in IgG4‐RD, which may mimic malignancy. In atypical presentations such as those occurring in older adults without conventional antibody positivity or with concurrent pancreatitis on imaging, clinicians should promptly consider early biopsy with immunostaining to avoid diagnostic delay and enable timely steroid treatment.

## 1. Introduction

Autoimmune hepatitis (AIH) classically presents in a middle‐aged female with hepatocellular enzyme elevation, hypergammaglobulinemia, interface hepatitis on histology, and antinuclear (ANA) and/or antismooth muscle (ASMA) antibodies although seronegativity may occur in atypical presentations [1, 2]. IgG4‐related disease (IgG4‐RD) is an uncommon multisystem fibroinflammatory disease with varying organ manifestations [3]. We report an older man with ANA/ASMA‐negative hepatitis, elevated serum IgG4, IgG4‐positive plasma cell–rich interface hepatitis on biopsy, and concomitant pancreatitis on imaging, consistent with IgG4‐related autoimmune hepatitis (IgG4‐AIH) with probable Type 1 autoimmune pancreatitis (AIP). This case is notable for CA 19‐9 > 1000 U/mL in the setting of pancreatitis and severe jaundice, initially raising concern for malignancy and other causes of abnormal liver enzymes (drug‐induced liver injury and iron overload).

## 2. Case Presentation

**FIGURE 1:**
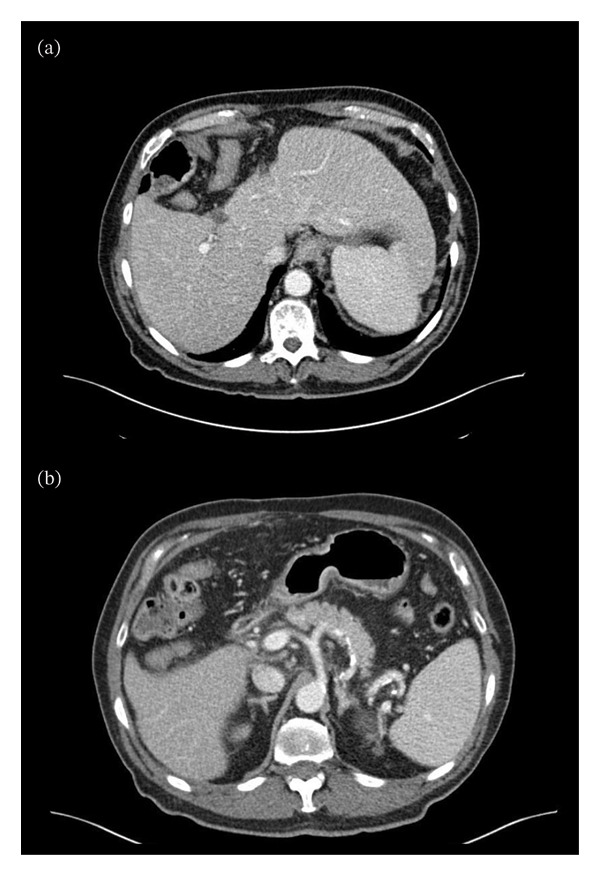
Computed tomography (CT) (image (a)) demonstrates minor hepatomegaly extending to the left upper quadrant. No hepatic lesions are present. Spleen size is at the upper limit of normal to mildly enlarged. Image (b) shows peripancreatic fat stranding and edema consistent with acute pancreatitis.

A man in his 80s with past medical history of Type 2 diabetes, hypertension, hypothyroidism, remote cholecystectomy, and mild cognitive impairment was hospitalized at an outside facility for abdominal pain approximately 3 weeks before being evaluated at our tertiary center. Computed tomography (CT) imaging (Figure 1) revealed minor hepatomegaly, mild infiltration of peripancreatic fat, and small volume pelvic ascites. At that hospitalization, laboratory studies were notable for a serum lipase of 135 U/L (< 61 U/L), total bilirubin 3.1 mg/dL, alanine aminotransferase (ALT) 589 U/L, aspartate aminotransferase (AST) 724 U/L, and alkaline phosphatase (ALP) 168 U/L, consistent with acute hepatocellular injury and hyperbilirubinemia. Initial infectious, toxic, and autoimmune serologies including ANA, ASMA, liver–kidney microsome IgG antibody, and testing for viral hepatitis were negative. Additional tests disclosed a prominently elevated CA 19‐9 of 1268 U/mL (< 36), total IgG 2166 mg/dL (700–1600), and IgG4 level of 442.5 mg/dL (3.9–86.4).

Hepatic ultrasonography demonstrated patent vasculature without biliary stones or dilatation. Magnetic resonance cholangiopancreatography (MRCP) showed a 5‐6 mm incidental pancreatic cystic lesion in the posterior uncinate process without biliary obstruction or dilatation; no hepatic contour abnormalities were noted, and the spleen size measured at the upper limit of normal to mildly enlarged on imaging. His abdominal pain gradually improved with supportive care, and he was discharged from that facility with joint diagnoses of acute pancreatitis and apparent drug‐induced liver injury (DILI) attributed to an over‐the‐counter laxative (product name and ingredients could not be confirmed due to patient recollection).

Eight days after discharge, the patient was instructed to return to the local emergency department after outpatient hospital follow‐up laboratories showed AST 877 U/L, ALT 383 U/L, total bilirubin 9.5 mg/dL, direct bilirubin 6.4 mg/dL, and albumin 2.8 g/dL (3.5–5.0). No leukocytosis was present. There was no encephalopathy, and with international normalized ratio (INR) 1.3 (0.8–1.1), the patient did not meet criteria for acute liver failure despite significant hyperbilirubinemia. He was readmitted to the hospital for worsening liver dysfunction.

**FIGURE 2:**
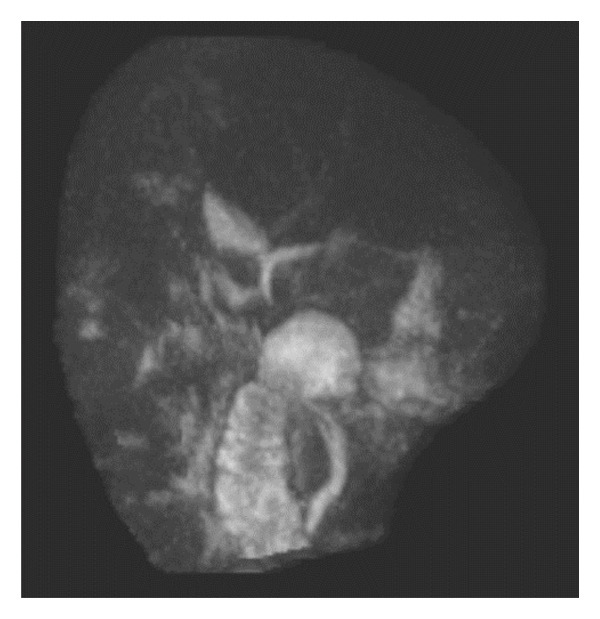
Coronal magnetic resonance cholangiopancreatography (MRCP) showed a surgically absent gallbladder without intrahepatic biliary dilation. The common bile duct was normal in course and caliber without filling defect.

CT abdomen/pelvis and MRCP (Figure 2) again demonstrated findings consistent with acute pancreatitis without biliary obstruction or mass, similar to prior imaging. CA 19‐9 decreased from 1268 U/mL to 851 U/mL. Viral hepatitis testing was again negative. Alternative etiologies were evaluated (ceruloplasmin, alpha‐1 antitrypsin, and phosphatidylethanol), all of which were unrevealing. Iron studies showed a transferrin saturation (TSAT) of approximately 92% (15–57), ferritin 1438 ng/mL (30.3–565.7), serum iron 182 μg/dL (41–186), and total iron binding capacity (TIBC) < 199 μg/dL (232–386). The patient underwent CT‐guided percutaneous liver biopsy and was then transferred to our hospital for evaluation of worsening jaundice and hepatocellular injury.

On interview, he denied abdominal pain but endorsed months of generalized pruritus. There was no family history of autoimmune, liver, or hematologic diseases. He reported a history of regular blood donation over many years as did his adult child. He denied symptoms suggestive of hemochromatosis, and review of available hospital records confirmed no recent blood transfusions. There was no use of new medications, supplements, or herbs. He denied alcohol or tobacco consumption. Physical exam revealed a jaundiced older man with normal vital signs and a distended, nontender abdomen without palpable hepatosplenomegaly. There was no encephalopathy. C‐reactive protein (CRP) was elevated (2.9 mg/dL; reference < 0.9 mg/dL). Lipase remained above the upper limit of normal at 104 U/L. Iron studies were similarly elevated compared with prior values, prompting concern for iron overload, although these indices can be distorted during acute hepatitis and systemic inflammation [4]. While biopsyunderwent pathologist review, HFE gene analysis revealed compound heterozygosity for the C282Y and H63D variants.

Approximately 3 weeks following his first hospitalization, liver biopsy uncovered preserved hepatic architecture with dense inflammatory infiltrates composed of lymphocytes, clusters of plasma cells, and neutrophils (Figure 3(a)). Numerous acidophil bodies were seen without significant steatosis. Bile ducts showed epithelial disarray without significant destruction or loss. Staining for iron (Figure 3(b)) showed focal (1+/4) hepatocellular iron deposition. IgG4 immunostaining demonstrated increased numbers of IgG4‐positive plasma cells up to 58 per high‐power field (shown in Figure 3(c)). No significant fibrosis was noted. Periodic acid‐Schiff with Diastase (PAS‐D) stain was unremarkable.

FIGURE 3(a) Liver specimen (hematoxylin and eosin stain, 100x magnification) reveals dense inflammatory infiltrates consistent with interface hepatitis. An interlobular bile duct (yellow arrow) shows epithelial disarray without destruction. The lobular parenchyma shows acidophil bodies (black arrows) without significant steatosis. (b) Iron stain (shown in blue, 100x magnification) with minor hepatocellular iron deposition. (c) Staining for IgG4 (200x magnification) displays increased numbers of IgG4‐positive plasma cells up to 58 per high‐power field.

**Figure:**
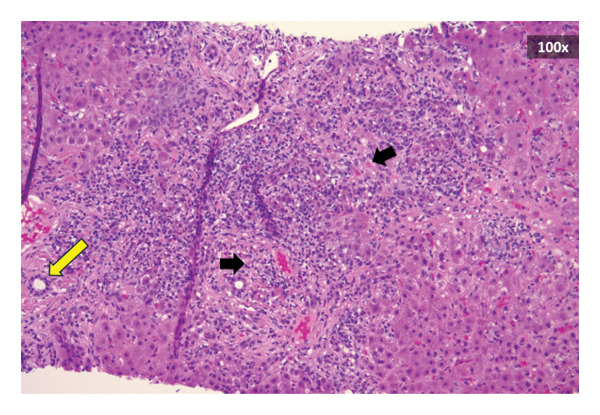
(a)

**Figure:**
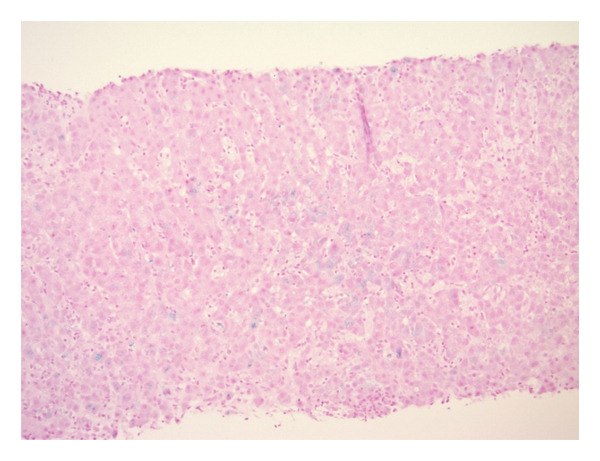
(b)

**Figure:**
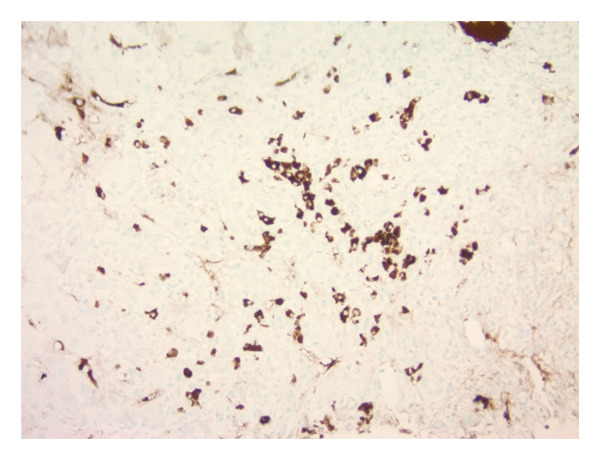
(c)

Although HFE compound heterozygosity can infrequently cause iron overload, the minimal iron deposition on biopsy, absence of typical hemochromatosis symptoms, and temporal association with acute hepatitis suggested the iron study abnormalities were secondary to inflammation rather than primary hemochromatosis. The results made DILI from the over‐the‐counter laxative unlikely and repeat iron studies after the resolution of current illness were ordered to ensure continued decline. He was diagnosed with IgG4‐AIH. Concomitant Type 1 AIP was suspected before prednisone initiation based on elevated serum IgG4, acute pancreatitis on imaging, and extrapancreatic organ involvement.

Because pancreatic tissue was unavailable and imaging findings were not pathognomonic, the pancreatic process was classified as probable Type 1 AIP after subsequent clinical review and steroid responsiveness, consistent with the International Consensus Diagnostic Criteria [5]. No additional manifestations of IgG4‐RD were noted. Follow‐up surveillance imaging for the incidental lesion with contrast‐enhanced MRI or pancreas‐protocol CT was advised. He was placed on oral prednisone 40 mg daily with plans for an extended course followed by gradual taper. Given anticipated prolonged steroid therapy, trimethoprim–sulfamethoxazole prophylaxis for *Pneumocystis jirovecii* pneumonia was initiated while prednisone dose remained ≥ 20 mg/day for ≥ 1 month, and cholestyramine was prescribed for pruritus. The patient was discharged home.

Interim laboratory testing on Day 12 of prednisone therapy showed early biochemical improvement, with decreases in aminotransferases and bilirubin compared with pretreatment values. Reassessment 26 days after treatment noted continued improvement. Repeat IgG4 level substantially decreased to 258.3 mg/dL, INR normalized, CRP declined to < 0.3 mg/dL, and liver enzymes began to normalize. On Day 32 of therapy, testing showed continued improvement in liver enzymes and hyperbilirubinemia. Repeat iron studies on Day 41 of prednisone therapy showed that ferritin decreased from 1438 to 863 ng/mL and TSAT decreased from approximately 92% to 69.5%, likely consistent with reduction in inflammation. Pruritus resolved and there were no apparent adverse events from treatment. By Day 57 of prednisone therapy, the patient had complete resolution of hyperbilirubinemia and normal transaminases, indicating resolution of acute illness.

Oral prednisone was continued at a taper of 35 mg daily for 30 days with future reassessment of dose and duration pending clinical stability. A slow taper was selected due to cholestatic severity, IgG4‐RD relapse concern, and outpatient logistics. Long‐term management was ultimately constrained by goals of care with limited follow‐up due to progressive debility and transfer to a skilled nursing facility. Additional invasive evaluation was deferred in alignment with patient goals of care.

## 3. Discussion

**FIGURE 4:**
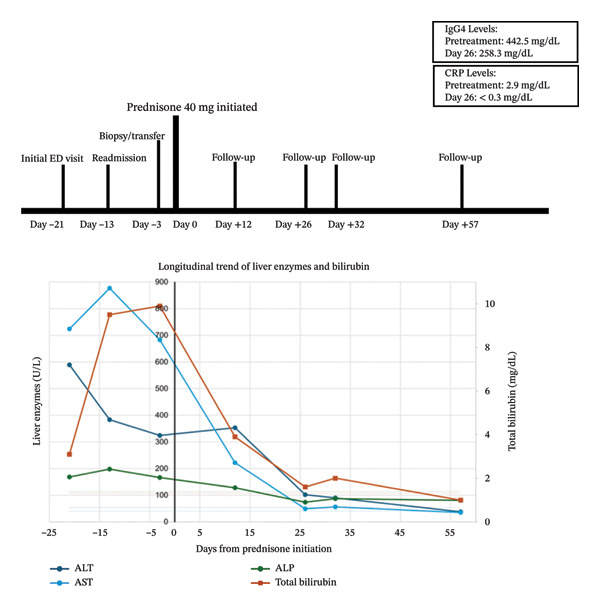
Timeline and trend of biochemical response to steroid therapy. Timeline of major events in patient clinical course with Day 0 (solid vertical line) corresponding to prednisone initiation. Trends in alanine aminotransferase (ALT), aspartate aminotransferase (AST), and alkaline phosphatase (ALP) are shown relative to the left y‐axis in U/L. Total bilirubin is displayed relative to the right y‐axis in mg/dL. Horizontal dashed lines represent the upper limit of normal for each parameter. C‐reactive protein (CRP) and IgG4 levels are displayed in the upper‐right inset. An improvement in variables after steroid treatment is apparent, with normalization of transaminases and total bilirubin by Day 57.

This case describes ANA/ASMA‐negative IgG4‐AIH and concomitant pancreatitis presenting with CA 19‐9 > 1000 U/mL and abnormal iron indices that improved as clinical inflammation and hepatitis resolved during steroid therapy. The patient was found to have elevated serum IgG4, interface hepatitis with dense IgG4‐positive plasma cell infiltrates (up to 58/high‐power field), and concomitant acute pancreatitis. Complicating a complex clinical picture, HFE testing for a considerably elevated ferritin and TSAT revealed compound heterozygosity for the C282Y and H63D variants. The patient was diagnosed with IgG4‐RD involving the liver, with probable Type 1 AIP, and improved with glucocorticoid therapy. Figure 4 displays a timeline of events.

Classical autoimmune hepatitis (AIH) typically affects middle‐aged females and presents with elevated aminotransferases, hypergammaglobulinemia, and positive ANA or ASMA serologies [6]. Most patients with IgG4‐AIH are female (up to 80% in some samples), though this patient diverged from the typical phenotype, being male, older, and seronegative [7]. In IgG4‐RD, conventional autoantibody negativity poses a diagnostic challenge and demonstrates the importance of obtaining IgG4 levels and pathologic specimen for review [3, 7].

This case strongly supports IgG4‐RD under the 2020 comprehensive diagnostic criteria, including serum IgG4 > 135 mg/dL, histopathologic findings of IgG4+ plasma cells > 10/high‐power field, and involvement of more than one organ system [2, 8]. Classic pathological findings such as storiform fibrosis or obliterative phlebitis were absent in this patient [2, 8]. The liver biopsy findings in this case were consistent with IgG4‐AIH, a rare subset of AIH previously described in the literature [2]. Histologically, IgG4‐AIH often shows lobular hepatitis, plasma‐cell rich infiltrates, and interface activity but may lack the classic storiform fibrosis and obliterative phlebitis seen in other organs [2, 9].

While AIH typically presents with significant elevations in serum aminotransferases, this patient also displayed significant conjugated hyperbilirubinemia despite the absence of biliary obstruction or mass‐forming pancreatitis, which has been reportedin similar case presentations [10, 11]. In this setting, jaundice was presumed to reflect severe hepatocellular injury with impaired bilirubin handling rather than a mechanical obstructive process [12, 13].

Imaging findings in AIH are highly variable; therefore, imaging has little role in diagnosis of IgG4‐AIH beyond excluding alternative conditions [14, 15]. Notable CT and MR imaging findings in this patient included minor hepatosplenomegaly and small volume pelvic ascites, without significant abnormalities in liver contour, morphology, or evidence of complications (e.g., portal vein thrombus). AIH may present with alterations in liver enhancement secondary to hepatocellular damage in approximately one‐third of the cases though normal homogeneous enhancement was present in this case [14].

This individual displayed concurrent mild pancreatitis most suggestive of Type 1 AIP as a manifestation of IgG4‐RD. Type 1 AIP commonly affects men above 50 years old and is often referred to as IgG4‐related pancreatitis due to its close association with IgG4‐RD as in this case. The International Consensus Diagnostic Criteria for AIP include a combination of clinical, serological, histological, imaging, and laboratory findings. The condition frequently presents with only mildly elevated serum lipase as in this patient [5, 16, 17].

AIP was considered probable rather than definite because imaging findings (Figures 1 and 2) were not pathognomonic. MRCP findings have been described as highly variable in AIP which may explain this discrepancy [18]. However, CT revealed findings consistent with pancreatitis and the patient displayed serum lipase elevation with abdominal pain. Although no pancreatic tissue was available for definitive confirmation, this patient’s elevated IgG4 level, imaging findings of pancreatitis, extrapancreatic organ manifestations, and response to steroids (noted retroactively) supported a diagnosis of probable Type 1 AIP.

This case included co‐occurrence of HFE heterozygosity with abnormal iron studies although the patient did not meet full phenotypic criteria for hereditary hemochromatosis. The significantly elevated TSAT and decreased TIBC are suggestive of iron overload [4]. In this individual, minimal hepatic iron staining and improving iron indices at follow‐up made true systemic iron overload unlikely. In the context of the patient’s IgG4‐RD with hepatic and pancreatic involvement, inflammation‐driven TSAT elevation was considered most likely. While HFE (C282Y/H63D) heterozygosity is fairly common and typically does not result in true hemochromatosis (low penetrance), it remains unclear whether iron overload could modulate immune‐mediated liver injury and a “double‐hit” phenomenon from co‐occurring inflammation and genetic factors warrants further investigation [19, 20].

Elevations in CA 19‐9 have previously been described in Type 1 AIP but are often lower (∼180–350 U/mL) than in this individual [21, 22]. CA 19‐9 was significantly elevated (1268 U/mL) and declined before steroid initiation (851 U/mL), suggesting a dynamic process rather than progressive malignancy (often confused with AIP) [5]. Notably, prior authors have reported elevation in CA 19‐9 with IgG4‐RD and associated inflammation [23, 24]. However, CA 19‐9 was not rechecked after clinical recovery, limiting conclusions about mechanism. In similar presentations, persistent elevation after resolution of cholestasis/pancreatitis should prompt renewed malignancy evaluation. If follow‐up was feasible, we would repeat CA 19‐9 after bilirubin normalization and consider endoscopic ultrasound if elevated or if lesion characteristics changed on follow‐up imaging if in line with the patient’s goals of care.

Corticosteroids remain the mainstay of treatment for IgG4‐RD, AIH, and AIP [16, 25, 26]. Long‐term relapse risk in this patient remains unknown given limited follow‐up; however, rapid biochemical improvement with glucocorticoids and maintained remission on low‐dose therapy is described in similar cases [10, 25]. Limitations of this report include the absence of pancreatic tissue diagnosis, unavailability of the IgG4/IgG plasma cell ratio on histopathologic review, no posttreatment CA 19‐9 measurement, and limited long‐term relapse surveillance after transfer to a skilled nursing facility.

## 4. Conclusion

This case presents a seronegative older adult with histologic and serologic features of IgG4‐RD involving the liver (IgG4‐AIH) with probable Type 1 AIP. IgG4 testing should be considered when evaluating hepatocellular injury or unusual pancreatitis presentation. Patients should be evaluated for extrahepatic manifestations if IgG4‐RD is suspected. Absence of ANA/ASMA positivity does not preclude the diagnosis of AIH. Elevations in CA 19‐9 may be seen in IgG4‐RD and AIP. Steroid treatment appears effective with strong potential for improvement after several months.

## Author Contributions

H.S.: conceptualization, methodology, investigation, data curation, writing–original draft, writing–review and editing, visualization, and supervision. S.R.: conceptualization, investigation, data curation, writing–original draft, writing–review and editing, and visualization.

## Funding

No funding was received for this study.

## Disclosure

All authors have read and approved the final version of the manuscript.

## Consent

Informed patient consent was obtained for publication of the case details.

## Conflicts of Interest

The authors declare no conflicts of interest.

## Data Availability

Data sharing is not applicable to this article as no datasets were generated or analyzed during the current study.
